# Unfinished business: A grounded theory analysis of change among individuals classified as numerical non‐responders to psychodynamic psychotherapy for post‐traumatic stress disorder related to childhood maltreatment

**DOI:** 10.1111/papt.70040

**Published:** 2026-02-18

**Authors:** Fatima Nöske, Frederike Döring, Manfred E. Beutel, Melissa Hitzler, Jürgen Hoyer, Elena Kabbathas, Christine Knaevelsrud, Iris‐T. Kolassa, Johannes Kruse, Falk Leichsenring, Nina Marin, Helen Niemeyer, Simone Salzer, Karoline Sauer, Marie Siebert, Rudolf Stark, Visal Tumani, Kerstin Weidner, Jörn von Wietersheim, Wolfgang Wöller, Christiane Steinert

**Affiliations:** ^1^ International Psychoanalytic University Berlin Berlin Germany; ^2^ Department of Psychotherapy and Psychosomatics Justus Liebig University Gießen Gießen Germany; ^3^ Department of Psychosomatic Medicine and Psychotherapy University Medical Center Mainz Mainz Germany; ^4^ Department of Clinical and Biological Psychology Institute of Psychology and Education Ulm University Ulm Germany; ^5^ Institute of Clinical Psychology and Psychotherapy Technische Universität Dresden Dresden Germany; ^6^ Division of Clinical Psychological Intervention, Department of Education and Psychology Freie Universität Berlin Berlin Germany; ^7^ Department of Psychotherapy and Psychosomatics Philipps University Marburg Marburg Germany; ^8^ Department of Psychosomatics and Psychotherapy University of Rostock Rostock Germany; ^9^ Department of Clinical Psychology, Psychotherapy and Experimental Psychopathology Johannes Gutenberg University Mainz Mainz Germany; ^10^ Center for Mind, Brain and Behavior Justus Liebig University Gießen and Philipps University Marburg Gießen and Marburg Germany; ^11^ Department of Psychotherapy and Systems Neuroscience Justus Liebig Universität Gießen Gießen Germany; ^12^ Department of Psychiatry Ulm University Ulm Germany; ^13^ Department of Psychotherapy and Psychosomatic Medicine, Faculty of Medicine and University Hospital Carl Gustav Carus TUD Dresden University of Technology Dresden Germany; ^14^ Department of Psychosomatic Medicine and Psychotherapy Ulm University Medical Center Ulm Germany; ^15^ Independent Advisor Bonn Germany

**Keywords:** childhood maltreatment, grounded theory, numerical nonresponse, patient perspective, PTSD, qualitative, trauma‐focused therapy

## Abstract

**Objectives:**

Individuals with post‐traumatic stress disorder related to childhood maltreatment (PTSD‐CM) show limited response to treatment on pre‐post‐symptom measures. While a nuanced understanding of nonresponse is crucial for improving treatment, quantitative measures may not fully capture clinically meaningful processes of change from the patients' perspectives. We therefore explored how individuals with PTSD‐CM who showed no or limited numerical improvement after trauma‐focused psychodynamic psychotherapy (TF‐PDT) experienced change.

**Design:**

This qualitative study was embedded in a large randomized controlled trial on PTSD‐CM (DRKS00021142).

**Method:**

From 75 qualitative post‐treatment interviews, we purposively sampled eight TF‐PDT recipients meeting criteria for numerical nonresponse, defined as current PTSD diagnosis and less than 50% reduction in the Clinician‐Administered PTSD Scale for DSM‐5 (CAPS‐5). Client Change Interviews were analysed using Critical‐Constructivist Grounded Theory.

**Results:**

We derived two clusters (‘Progress—A Double‐Edged Sword’, ‘When Building Trust Collides with the Therapeutic Framework’) and one overarching core category (‘Unfinished Business’), which captured a dialectic process. Within a responsive therapeutic relationship, patients began confronting their relational trauma, experiencing relief, increased emotional regulation and hope. However, this very engagement surfaced emotional challenges that exceeded what could be addressed within the limited therapeutic framework, leaving the process meaningful but unfinished.

**Conclusion:**

What is often labelled as numerical nonresponse in PTSD‐CM may reflect a dynamic interplay of emerging improvement and emotional distress that needs more time to unfold. Moreover, the results underscore the value of integrating qualitative, patient‐reported outcomes into treatment evaluation and relationship‐focused treatment tailored to patients' individual needs.

## INTRODUCTION

Childhood maltreatment (CM)—that is, the emotional, physical and sexual abuse and/or neglect of individuals under the age of 18—is a serious global concern (Hillis et al., 2016; Sethi et al., 2013). The effects of CM often persist into adulthood, for example, increasing the risk for unemployment (Witt et al., 2017), revictimization (Fereidooni et al., 2023), mental problems (Teicher et al., 2022), and high healthcare costs (Grochtdreis et al., 2025). A common consequence, especially after physical or sexual abuse (Gilbert et al., 2009), is post‐traumatic stress disorder (PTSD; International Classification of Diseases and Related Health Problems [ICD‐11], World Health Organization [WHO], 2019). PTSD related to childhood maltreatment (PTSD‐CM) often includes a more complex symptomology, such as a negative self‐concept, interpersonal difficulties or affective dysregulation. These features may meet diagnostic criteria for complex PTSD (CPTSD), as defined by ICD‐11 (WHO, 2019). Trauma‐focused therapies are recommended as first‐line treatments for PTSD(‐CM), with strong evidence for exposure‐based cognitive‐behavioural therapy (TF‐CBT; Hamblen et al., 2019). Interpersonal and psychodynamic approaches, though less extensively studied, are commonly used and seem to be effective as well (Keefe et al., 2024). One such approach is trauma‐focused psychodynamic therapy (TF‐PDT; Wöller et al., 2012, 2020), which integrates psychodynamic principles with imaginative techniques to strengthen ego functioning and internal resources. Quasi‐experimental studies and randomized controlled trials (RCTs) support its effectiveness (Brom et al., 1989; Lampe et al., 2024; Levi et al., 2016; Riedl et al., 2025; Sachsse et al., 2006; Steinert et al., 2017). Despite trauma‐focused therapy being first‐line treatment for PTSD, non‐response^1^ rates can reach up to 50% (Cuijpers et al., 2024; Schottenbauer et al., 2008), and individuals with PTSD‐CM may be especially vulnerable (Cloitre et al., 2010). Yet non‐response is less studied than deterioration or treatment success (Krivzov et al., 2021; Lambert, 2011; Paveltchuk et al., 2022). Existing group‐level predictors like comorbid depression, male gender, and older age (Semmlinger et al., 2024) may not generalize to individual cases (Truijens et al., 2019). Also, quantitative outcome measures may not capture what changes from the patients' perspective are considered meaningful or what hinders such changes (Kazdin, 2006; Ladmanová et al., 2022, 2024; Vybíral et al., 2024). Qualitative systematic reviews highlight multiple hindering factors in trauma‐related therapy from the patients' perspective, such as dissatisfaction with the intervention, external burdens, or a negative therapeutic relationship (Dawood et al., 2025; Lepistö et al., 2025). Additionally, mixed‐method studies show that individuals who did not improve according to quantitative measures may experience therapy as helpful, suggesting that numerical non‐response does not necessarily indicate the absence of therapeutic effect (McElvaney & Timulak, 2013; Werbart et al., 2015). Carrington et al. (2024) conclude that these discrepancies point to ‘flaws in the way therapeutic outcome is conceptualized and measured’ (p. 12). These limitations have prompted growing calls for involving individuals with lived experience and integrating qualitative approaches into psychotherapy research (Bartholomew & Lockard, 2018; Davis et al., 2024; Kazdin, 2022; Levitt et al., 2021; Lutz & Hill, 2009). These efforts can deepen understanding of therapeutic change, refine interventions (Levitt, 2018; Levitt et al., 2016; Maxwell & Levitt, 2023) and address power imbalances in how knowledge and interventions are developed (Davis et al., 2024).

### How do numerically defined non‐responders’ experience therapy?

Qualitative and mixed‐method studies offer insight into the discrepancies between ‘non‐response’ based on commonly used pre–post‐treatment measures and the individuals’ subjective experience. In their meta‐synthesis, Carrington et al. (2024) found that individuals classified as ‘non‐responders’ across various mental disorders reported partial improvements and hope for further gains, alongside dissatisfaction with treatment and disconnection in the therapeutic relationship. Other studies further demonstrate the nuanced therapy experience of ‘non‐responders’ with depressive and anxiety disorders. They described feeling stuck, struggling to implement positive change, experiencing their therapist as passive or avoidant, and therapy as superficial or unstructured, alongside burdens such as comorbidities and time‐limited treatment (De Smet et al., 2019; Mehta et al., 2023; Werbart et al., 2015; Westra et al., 2010). At the same time, many also reported subjective benefits such as greater emotional stability, insight, improved relationships and renewed hope, often linked to a nonjudgmental and validating therapeutic stance (De Smet et al., 2019; MacLeod, 2017).

To our knowledge, only two studies have specifically examined the lived experience of CM‐survivors in relation to numerical outcome: Young adults classified as ‘non‐responders’ according to quantitative measures reported increased well‐being and meaningful engagement with past trauma, alongside persistent symptoms such as sleep problems or fragmented memories. A triggering environment was identified as a key barrier for treatment effectiveness (Onsjö et al., 2025). However, the study took place several years post‐treatment, limiting insight into patients’ experience immediately after treatment. Olofsson et al. (2020) identified unaddressed trauma and a negative therapeutic relationship as factors hindering improvement among individuals with eating disorders and CM after completing a CBT or compassion‐focused treatment for eating disorders. However, since the study addressed eating disorders in a non‐trauma‐focused therapy, its findings cannot be applied to treatment approaches for PTSD‐CM. Also, because quantitative data were assessed one year after treatment, unknown factors may have influenced participants’ posttreatment trajectories (Olofsson et al., 2020).

Taken together, these findings show that numerical ‘non‐response’ does not necessarily mean that no change has occurred. Defining individuals as ‘non‐responders’ based on quantitative pre‐post‐outcome measures may overlook their nuanced experience, such as subtle improvements or potential reasons for non‐improvement, highlighting the need to complement quantitative symptom‐focused outcomes with qualitative accounts from the patients’ perspective.

### Aim of the present study

Despite the high prevalence of non‐response in PTSD‐CM, no study to date has qualitatively explored whether and how individuals classified as ‘non‐responders’ experienced change when interviewed right after trauma‐focused treatment. Addressing this gap, the present study examines the following question: *How did individuals with PTSD‐CM who showed little or no numerical improvement in the primary outcome measure following TF‐PDT experience change, and which factors facilitated or hindered this process?* We aim to enhance the understanding of non‐response in PTSD‐CM from diverse perspectives and in greater depth by employing a qualitative and explorative approach. Since our study is embedded in the first RCT to examine the efficacy of a *manualized* TF‐PDT (Wöller et al., 2020) in PTSD‐CM, we also aim to inform further development and implementation of TF‐PDT.

## MATERIALS AND METHODS

### Setting and sample

This study was embedded in the ENHANCE research network (ENHANCE; Leichsenring et al., 2020), funded by the German Federal Ministry of Education and Research (trial registration number: DRKS00021142), with the focus on enhancing the understanding and treatment of PTSD‐CM. The non‐inferiority RCT in ENHANCE is the first trial comparing TF‐PDT (Wöller et al., 2012, 2020) and skills training in affective and interpersonal regulation (STAIR) narrative therapy (SNT; STAIR‐NT; Cloitre et al., 2002, 2020), a form of TF‐CBT, for adults with PTSD‐CM. More than 360 eligible participants were randomized to STAIR‐NT, TF‐PDT or a control group (minimal attention waiting list) in 10 outpatient centres (5 STAIR‐NT/5 TF‐PDT) in five participating German cities. Participants were adults of all genders aged 18–65 with a primary diagnosis of PTSD due to CM before age 18. Treatment in both conditions consisted of up to 24 weekly sessions. Exclusion criteria included current psychotic disorders, ongoing abuse, acute suicidality, acute substance dependence, borderline personality disorder, dissociative identity disorder, organic mental disorders, severe medical conditions, newly initiated pharmacotherapy or concurrent psychotherapy. The primary outcome was assessed via the Clinician‐Administered PTSD Scale for DSM‐5 (CAPS‐5; Müller‐Engelmann et al., 2020) by trained and blinded raters. The project Patient Participation is a qualitative sub‐project in ENHANCE and focuses on helpful and hindering aspects of psychotherapy from the patients' perspective (funding ID: 01KR1801D). Each of the 10 centres used a random number generator to select 7–8 participants from both treatment arms who received at least 10 therapy sessions for potential interviews. Selection was independent of treatment outcome. Participation was voluntary, and if someone declined, the next eligible participant was approached. Ultimately, 75 patients were interviewed. Ethical approval was obtained for both ENHANCE and Patient Participation; participants provided informed written consent, and data were securely stored in accordance with ethics committee regulations.

### Trauma‐focused psychodynamic therapy (TF‐PDT)

TF‐PDT is a manualized treatment for trauma‐related disorders following CM, grounded in psychodynamic theory (Wöller et al., 2012, 2020). According to the associative model underlying TF‐PDT (Wöller, 2016; Wöller et al., 2020), children exposed to ongoing caregiver violence often split off these traumatic experiences into a separate state of consciousness. This defense mechanism protects them from overwhelming affect and helps preserve the positive caregiver representations needed for psychological survival. However, trauma‐associated triggers can later reactivate these intrusive and highly distressing fragments. TF‐PDT aims to reduce this distress by re‐associating these dissociated fragments with positive self‐ and object representations. It adopts an active therapeutic stance with limited regression and prioritizes a secure therapeutic relationship, patient control, transparency and explicit consent. The treatment model also acknowledges that PTSD‐CM is connected to complex transference and countertransference phenomena like projective identification or perpetrator and rescuer transferences (Wilson & Lindy, 1994). Accordingly, the psychodynamic approach emphasizes recognizing and managing one's own countertransference reactions in order to provide a secure emotional presence and maintain reliable therapeutic boundaries. TF‐PDT is structured into three phases that are flexibly adapted to patients' needs. The **s**tabilization phase aims to enhance emotion regulation and ego functions related to self and relationships through resource activation and imaginary techniques like the ‘safe place’ (Wöller et al., 2012, p. 80). When the patient has achieved sufficient emotional regulation and stability in everyday activities, a gradual and gentle trauma confrontation phase may be initiated, adopting techniques like the ‘screen technique’ (Wöller et al., 2012, p. 84). The integration phase addresses interpersonal difficulties, new challenges, and prepares for therapy termination. Ultimately, psychodynamic techniques like confrontation, clarification and interpretation may be used to work through conscious and unconscious conflicts. Although TF‐PDT outlines treatment phases, they serve as a flexible framework rather than a fixed sequence. Consistent with psychodynamic principles, therapists move between phases according to the patient's needs, clinical indication, and the evolving intrapsychic and relational dynamics. Moreover, trauma confrontation is not mandatory in TF‐PDT.

### Therapists in TF‐PDT

All TF‐PDT therapists were psychologists or licensed physicians and had completed their psychodynamic psychotherapeutic training or were in advanced psychodynamic psychotherapeutic training. Before treating study participants, they underwent a two‐day training in TF‐PDT provided by the manual's main developer and treated one pilot case under supervision. Throughout the treatment period, ranging from 2020 to 2024, therapists were supervised biweekly by TF‐PDT manual experts and participated in additional training. To assess therapists' adherence and competence in delivering treatment, all sessions were audio‐recorded. Three sessions per case were randomly selected from the early, middle and late treatment phases and were rated by trained, masked evaluators using treatment‐specific checklists.

### Participants of the present study

From the 75 interviewed participants in Patient Participation, we purposefully sampled individuals with direct experience relevant for our research question (Palinkas et al., 2015), that is, individuals who did not improve according to CAPS‐5 after TF‐PDT (so‐called ‘non‐responders’). Non‐response was defined as (a) current PTSD diagnosis and (b) a symptom reduction of less than 50% according to CAPS‐5. Our definition of non‐response aligns with the ENHANCE‐RCT (Leichsenring et al., 2020) and with previous systematic reviews on PTSD (Cuijpers et al., 2024; Varker et al., 2020). While other studies have used lower thresholds or reliable change indices (e.g., pre–post difference of 11 points on the CAPS‐5; Sele et al., 2023), no uniform definition of response or non‐response in PTSD exists (Cuijpers et al., 2024). Our choice ensured consistency with the ENHANCE‐RCT and allowed us to foreground the experiences of those categorized as ‘non‐responders’ within that framework. Based on our definition, we included eight participants in the present study. All self‐identified as female, aged 21 to 63 years (*M* = 48.25, SD = 14.32), with up to five comorbid disorders at baseline according to SCID‐5‐CV and SCID‐5‐PD (First et al., 2016). According to the self‐report measure International Trauma Questionnaire (ITQ, Cloitre et al., 2018), all but one participant met the criteria for CPTSD. For detailed characteristics, see Table 1.

**TABLE 1:** Participants‘ characteristics.

Participant number	Age at randomization	Prior experience with psychotherapy	Comorbid diagnosis	CAPS‐5 baseline	CAPS‐5 post	CPTSD baseline	Interview length (in minutes)
P1	32	No	Persistent depressive disorder, obsessive‐compulsive disorder, avoidant personality disorder	36	40	Yes	80
P2	52	Yes	Major depression, agoraphobia, social anxiety disorder	28	22	No	97
P3	21	No	Major depression, persistent depressive disorder, panic disorder, specific phobia	38	35	Yes	36
P4	57	Yes	Major depression, persistent depressive disorder, panic disorder, generalized anxiety disorder, somatic symptom disorder	41	42	Yes	77
P5	58	Yes	Major depression, agoraphobia, avoidant personality disorder, paranoid personality disorder	43	29	Yes	100
P6	53	Yes	Major depression, persistent depressive disorder, avoidant personality disorder, specific phobia, binge eating disorder	45	37	Yes	86
P7	63	Yes	Major depression	42	40	Yes	81
P8	50	Yes	Major depression, agoraphobia, attention‐deficit/hyperactivity disorder, avoidant personality disorder	40	36	Yes	60

*Note*: All participants were female; CPTSD diagnosis according to the International Trauma Questionnaire (ITQ).

### Qualitative interview

We used the semi‐structured Client Change Interview (CCI; Elliott, 1999; Elliott & Rodgers, 2008) that we translated and adapted for our study context. It encompassed questions about perceived changes, helpful and hindering aspects, personal strengths and weaknesses, the current life situation, and study participation. For the English version of the interview, see the Supporting Information. The trained interviewers encouraged participants to reflect freely on their experience, including any discomfort, uncertainties, or overlooked areas, to foster an open and respectful dialogue (Levitt, 2021). Time was reserved at the end for any topic participants felt had not yet been addressed. Interviews of the eight participants (range: 36–100 min, *M* = 77) were conducted between March 2022 and April 2024 in person, via telephone or via video call. Interviewers were not involved in the patients’ diagnostic assessment or treatment and were not aware of their numerical outcomes. Participants were also unaware they were classified as ‘non‐responders’. Additionally, they were informed that their therapists would not receive any feedback from the interviews. No financial compensation was offered. Interviews were audio‐recorded, pseudonymized and transcribed verbatim using the easytranscript software (John, 2021).

### Research team of the present study

The core team consisted of the first author (FN), a psychologist, doctoral student and psychodynamic trauma counsellor. The second author (FD), a graduate student, conducted the preliminary analysis as part of her master's thesis. The senior author (CS) is a professor of clinical psychology and psychotherapy, a licensed psychodynamic psychotherapist and the Principal Investigator of Patient Participation alongside FL. Further members of the core team were two doctoral students and a research associate who were psychodynamic psychotherapists or psychotherapists in training: MS, who interviewed most of the eight participants, and NM. EK, a research associate, transcribed most of the interviews. Additional contributors included ENHANCE centre directors or research associates who were involved in recruitment, diagnostics and supervision, seven with CBT and six with a psychodynamic background. WW led the TF‐PDT manual development, supervised therapists and served as external advisor.

### Data analysis

For the qualitative analysis of the interviews of the eight participants classified as ‘non‐responders’, we followed the Critical‐Constructivist Grounded Theory (GT; Levitt, 2021). GT is an inductive qualitative approach, particularly suitable for our research question, as it aims to develop an understanding of participants’ lived experiences while remaining grounded in the data. We did not apply pre‐defined theories or hypotheses. The critical and constructivist perspective emphasizes that meaning is co‐constructed and shaped by researchers' perspectives (Levitt, 2021; Thornberg & Charmaz, 2014). Rather than constructing a complete theoretical framework, we sought to develop an understanding of the research topic (Levitt et al., 2021). Transcripts were coded using Atlas.ti software and initially segmented into meaning units (open coding), maintaining proximity to participants’ original wording. Through constant and systematic comparison of these meaning units, we identified recurring patterns and derived initial categories. Then, by examining their similarities and connections (Strauss & Corbin, 1998), we organized them into higher‐order categories (axial coding), which we further grouped into clusters and ultimately synthesized into an overarching core category. To cross‐check our results, we included two additional TF‐PDT ‘non‐responders’ in the analysis. Since the ENHANCE RCT and the project PP were ongoing during our data collection, these individuals were interviewed at a later point in the study timeline and were therefore not part of the initial analysis. Once their interviews became available, they were selected based on the same predefined non‐response criteria (current PTSD diagnosis and <50% symptom reduction on the CAPS‐5). Their interviews did not provide any new content. After completing our final analysis, three further individuals meeting non‐response criteria were interviewed in the project PP. However, these interviews were not included in our analysis, as the two additional interviews yielded no new content. In total, we analysed 10 of the 13 TF‐PDT ‘non‐responders’ interviewed in PP—eight in the main analysis and two to cross‐check our findings.

The data analysis deepened our understanding of the underlying dynamics and broader meaning behind participants’ experiences. Reflexive memoing (Levitt, 2021) guided analysis, ensuring alignment with participants’ narratives and the research question. By triangulating quantitative and qualitative data, our aim was not to privilege one perspective over the other, but rather to deepen understanding of the phenomenon by integrating multiple perspectives to account for the complexity and richness of experience (Bailey‐Rodriguez, 2021).

### Reflexivity and methodological integrity

To foster reflexivity (Levitt, 2021), the core team discussed individual perspectives and assumptions regarding non‐response in PTSD‐CM. Given the core team's shared psychodynamic orientation and positive personal experiences with psychodynamic therapy, we acknowledged a possible bias in not detecting negative aspects in TF‐PDT. While paying attention to negative experiences to identify potential hindering aspects for progress, we aimed to approach and interpret the data openly, while being mindful of our expectations. Our expertise in psychodynamic therapy was both a strength and a potential bias, which CBT‐oriented co‐authors helped balance through critical feedback. Reflective memos were kept for each transcript with notes on impressions, emerging ideas and biographical context. This helped us form an initial impression and allowed us to stay close to the participant's narrative. Preliminary categories were refined collaboratively, ensuring a shared understanding of the data. We also addressed internal team hierarchies to ensure an inclusive and collaborative analytic process. Because of ethical guidelines, we could not obtain participant feedback on the results, but we revisited audio recordings and critically reflected on the data. Aware of power dynamics, we followed trauma‐informed principles (Karmakar & Duggal, 2024), treating participants as experts and the interviewer as an open‐minded listener and learner (Schleider, 2023).

## RESULTS

We derived one overarching core category (‘Unfinished Business’), which encompassed two clusters: ‘Progress—A Double‐Edged Sword’ and ‘When Building Trust Collides with the Therapeutic Framework’. These clusters each contained three categories, each encompassing up to seven subcategories (see Table 2). The core category, clusters and categories are described in greater detail below.

**TABLE 2:** Overview of clusters, categories and subcategories.

Cluster	Categories	Subcategories
**1 Progress**—**A double‐edged sword** A more nuanced perception of self and others facilitated symptom management and more fulfilling interpersonal relationships. At the same time, encountering trauma‐related experiences intensified emotional distress. (*n* = 8)	1.1 Enhanced self‐awareness fostered inner dialogue, enabling improved emotion regulation and a greater sense of control. (*n* = 8)	1.1.1 I have developed a clearer awareness of my emotions, needs and triggers, which helps me be more in touch with myself and become more self‐caring (*n* = 6) 1.1.2 I am better able to regulate overwhelming states through using inner resources, creative tasks or a secure container (*n* = 6) 1.1.3 Inner child work was emotionally challenging but helped me to care for my inner child selves without losing myself, for example, by providing my inner child selves a safe place (*n* = 6) 1.1.4 I feel more stable and organized in everyday life and find it easier to be alone without being overwhelmed by distressing material (*n* = 4) 1.1.5 I have regained some joy in life and no longer feel an urge to act on my suicidal thoughts (*n* = 4)
	1.2 Reflecting on relational patterns facilitated a more integrated perception of others, allowing for deeper intimacy and self‐protective boundaries. (*n* = 7)	1.2.1 I have realized that I tend to transfer experiences from traumatic situations to current ones. As I have learned to better distinguish the present from my traumatic past, I have become more open and optimistic (*n* = 3) 1.2.2 I have developed a more understanding and positive view of family members — or of myself (*n* = 4) 1.2.3 My behaviour is no longer primarily focused on pleasing others; I prioritize my own needs more and set clearer boundaries (*n* = 6) 1.2.4 In social interactions, I have developed a more emotionally balanced and engaged way of interacting, which has led to fewer escalations in conflict (*n* = 2) 1.2.5 Now I can allow for a bit more physical or emotional closeness (*n* = 5) 1.2.6 Even though I still have fears of being judged or attacked by others, I avoid going out and encountering people less often than I used to (*n* = 2) 1.2.7 I have been able to lower my high expectations of myself and others (*n* = 2)
	1.3 Being confronted with traumatic memories and feelings led to increased symptoms and painful yet meaningful realizations. (*n* = 8)	1.3.1 Bringing up my traumatic experiences has brought back terrible memories and triggered frequent nightmares or intense physical reactions (*n* = 4) 1.3.2 I have painfully realized that being left alone and not protected was one of the most devastating aspects of my traumatic experiences (*n* = 2) 1.3.3 I am no longer solely stuck in my emotionally numb functional mode; instead, I have felt sadness about what happened to me. Sometimes, I can even feel anger again (*n* = 3) 1.3.4 I have only been able to classify certain experiences as trauma and realize the extent of my traumatic experiences through therapy (*n* = 6) 1.3.5 I have been able to gain some distance from my feelings of guilt about my traumatic experiences (*n* = 2)
**2 When building trust collides with the therapeutic framework** A therapeutic attitude attuned to patients' needs was essential for meaningful engagement. However, the limited duration of therapy and a reserved therapeutic stance reactivated feelings of abandonment. Moreover, external stressors could not be integrated into the therapeutic work, thereby hindering progress. (*n* = 8)	2.1 Feeling prioritized in a non‐abusive therapeutic alliance was experienced as helpful, whereas a reserved therapeutic stance led to confusion and disappointment. (*n* = 8)	2.1.1 I was very relieved to have someone outside of my life to talk to who does not judge me (*n* = 3) 2.1.2 For building trust, it was very important for me not to feel pressured and to be able to decide how the therapy would proceed (*n* = 4) 2.1.3 I felt a sense of safety because I experienced my therapist as confident and resilient, and it was helpful that they paid close attention to me and sometimes intervened in a regulating way (*n* = 6) 2.1.4 I was able to engage more easily when the therapist had a gender I could trust more because of my traumatic experiences. When I opened myself up to working with a male therapist despite my preference for a female therapist, I was able to have a helpful new relationship experience over the course of therapy (*n* = 6) 2.1.5 I have felt seen and taken seriously by the therapist (*n* = 7) 2.1.6 At times, my therapist was too passive; I would have wished for more active or more empathic support, especially when I wanted to share traumatic experiences but struggled to speak (*n* = 4)
	2.2 In light of the patients' complex trauma histories, therapy was considered too short. (*n* = 6)	2.2.1 Some of my interactional difficulties, such as hesitation or being unable to express myself, made the therapy process more difficult (*n* = 4) 2.2.2 At the beginning, I was afraid of the therapy. It took several sessions before I could trust enough to open up (*n* = 3) 2.2.3 The therapy ended in the middle of the process, and I felt left alone again. The prospect of follow‐up sessions or continued treatment made the end of therapy more bearable (*n* = 4) 2.2.4 The therapy period was too short to work through my issues. I would have needed the therapy to continue for longer (*n* = 6)
	2.3 External life stressors could not be integrated into trauma work, thereby hindering progress. (*n* = 6)	2.3.1 At times, I was additionally burdened by external conflicts and challenges. Occasionally, the therapy process was delayed due to these urgent issues (*n* = 3) 2.3.2 Conflicts with my family, my friends or a separation had a negative impact on my well‐being (*n* = 4)
**Core category: Unfinished business—The challenge of navigating relational trauma within a limited timeframe** Within a secure therapeutic relationship, patients took a risk and turned towards their trauma which brought relief and fostered a sense of hope that progress was possible. However, this very engagement with the traumatic past also led to new emotional challenges. Long‐standing issues, a reserved therapeutic stance and the limited timeframe hindered further progress. Ultimately, patients could only begin to lift the burden of their relational trauma.		

### CLUSTER 1: Progress—A double‐edged sword

**A more nuanced perception of self and others facilitated symptom management and more fulfilling interpersonal relationships. At the same time, encountering trauma‐related experiences intensified emotional distress.**

Participants reported improved self‐awareness, emotion regulation and relational functioning. However, engaging with traumatic memories and feelings was accompanied by emotional pain and intensified trauma symptoms. Despite the challenges, most viewed these changes as ultimately meaningful. All eight participants contributed to this cluster.

#### Category 1.1: Enhanced self‐awareness fostered inner dialogue, enabling improved emotion regulation and a greater sense of control

The interviewees described gaining a deeper understanding of their inner experiences, which supported both emotional regulation and a more compassionate relationship with themselves. Several reported recognizing for the first time how emotional distress manifested physically: ‘So, before … when I didn't feel good, I … went to the doctor …. But meanwhile, I wonder, is this actually something physical, or is it just because I'm really not feeling well?’ (P1). This increased awareness enabled them to identify personal needs and act on them more consciously. P5 stated that before therapy, ‘[I] didn't have the intuition that it bothers me, I just pushed it aside and then kept going’.

To support this process, participants used strategies from the TF‐PDT manual, including the construction of imaginative ‘container[s]’ (Wöller et al., 2012, p. 80), ‘safe place[s]’ (p. 80) or “inner child” work’ (p. 85). Although initially challenging, these techniques became effective tools for managing overwhelming emotions: ‘I just have to really… think about it [the safe place] and it's around me and I feel safer’ (P1). Another participant reported: ‘I have … [created a container] for the worst persons. With time I have managed to lock them away. The actions are still there. But the actions no longer harm me’ (P6).

In addition to imaginative techniques, participants used practical regulation strategies such as breathing techniques and consciously interrupting intrusive thoughts. Several discovered they could actively access positive emotions:

That it is really possible … not only to evoke feelings of anxiety and panic, but … also consciously to evoke positive feelings, that is … the biggest realization that I have gained there, that … comes often into play in my life, and what really makes me feel good. (P4)

Through becoming able to regulate trauma‐associated affects, participants gained greater control over their experiences. One participant described what it felt like to be alone, and how she experiences it now:

I've now understood that it's simply the fear of facing myself. And that has really improved. … I think that I'm no longer so afraid … that so much of the past will come up, but I can now be more in the here and now. (P2)

For some, these changes led to a revived sense of vitality, a stronger ‘will to live’ (P8) and more joy: ‘I laugh more often … I just have more fun in life’ (P2).

#### Category 1.2: Reflecting on relational patterns facilitated a more integrated perception of others, allowing for deeper intimacy and self‐protective boundaries

Participants described becoming aware of their relational patterns, such as submissiveness, avoidance, or verbal aggression, and recognized how these were shaped by their traumatic experiences. Several noticed how past experiences influenced current interactions: ‘You have long black hair, you can go. Just like that. Because I just had my bad experiences’ (P6). Another participant noted: ‘How old is this feeling, you know? This question was incredibly helpful to me because I then realized … this is an old feeling that I often experience, but it doesn't actually have much to do with reality’ (P2).

Through taking different perspectives, half of the interviewees developed a more integrated perception of others, even in difficult relationships associated with their traumatic experiences: ‘I can't forgive her [my mother] … but I can approach her with a little more kindness, because I know that she was or is ultimately just as traumatized as we are’ (P5).

Improved self‐perception and emotional regulation contributed to increased assertiveness, stopping saying ‘yes, of course’ (P1) to everything, but instead setting boundaries: ‘I'm no longer the plaything of others, but I also set a clear limit and say “ok, that's enough”’ (P5). Furthermore, some noticed becoming less ‘explosive’ (P5) in interactions:

So what's very … important for me … especially with my partner … is to have a way of dealing with it so that I don't always end up in a disaster, in alienation … I was extreme then … And that it doesn't happen anymore. (P2)

More than half reported overcoming avoidance in social situations and allowing more closeness in intimate relationships:

I've now visited my boyfriend's family, I avoided that before because I was too scared. … I'd already met the siblings last year, oh I was so tense. … I've also … had triggers and then had to cancel, so that was really rough. And now I was totally relaxed, I mean I was nervous, but I was able to approach them openly and I think that's really, really great! (P2)

Another participant described:

I've simply learned that I can let my husband come closer to me. Without anything happening to me. … He's now got to the point where he is allowed to hold me in his arms and give me a kiss. That was impossible! He *wasn't* [with emphasis] allowed to. … Now we always hug each other in the morning. And when he comes home from work, then too. And that feels good.

#### Category 1.3: Being confronted with traumatic memories and feelings led to increased symptoms and painful yet meaningful realizations

By addressing traumatic experiences, participants were confronted with distressing memories and feelings that they previously did not have access to. This led to intensified posttraumatic symptoms and to painful realizations, while also facilitating a more complete recognition of their traumatic past.

For half of the interviewees, confronting traumatic experiences during therapy led to several forms of distress, including nightmares or somatic reactions: ‘I always had quite a lot of pain, afterwards or even during [therapy] … I sometimes even had to vomit or got diarrhoea at home’ (P4). Another participant pointed out: ‘During this therapy, the part that I had packed away as a child came up again. … and that's why everything got worse and worse, because in the end, I could remember every detail again’ (P1). This process was summarized by P6 as particularly taxing: ‘The deeper you went into the details, the greater the stress afterwards’.

Confronting their trauma history was accompanied by painful realizations. Some participants realized that not the trauma itself but not being protected by the caregivers was particularly painful: ‘And what I also have underestimated … much worse, in fact much, much worse came up that my mum didn't protect me!’ (P7). The same participant reflected on how these insights opened access to emotional responses, such as sadness and anger: ‘I started crying. I'd hardly ever cried before. … It's like, as if someone has turned on a tap’ (P7). Eventually, facing the traumatic experiences led to a more complete understanding of their extent. Before therapy, some perceived certain forms of assault as trivial: ‘And when I said that I really don't know if it was an assault … And then the doctor said “*It was an assault. Definitely*” [with emphasis]. …I didn't even recognize it as such’ (P7).

Another participant concluded:

I actually already went there [to therapy] with the attitude that I knew what had happened. And that I was also able to explain certain aspects that resulted from it. But I was surprised in the sense that it apparently still took this time to actually accept it [the traumatic experiences]. (P3)

### CLUSTER 2: When building trust collides with the therapeutic framework

**A therapeutic attitude attuned to patients' needs was essential for meaningful engagement. However, the limited duration of therapy and a reserved therapeutic stance reactivated feelings of abandonment. Moreover, external stressors could not be integrated into the therapeutic work, thereby hindering progress.**

The helpful and hindering factors in this cluster had a profound influence on the patients' experience of their therapy and contributed to the changes described in Cluster 1. Participants highlighted a supportive therapeutic relationship based on their needs as a central factor in facilitating positive changes. Overall, they felt increasingly safe with their therapist. However, when those needs were not met, patients were not able to engage meaningfully with therapy. Ultimately, 24 sessions were not enough time to address long‐standing problems. All eight participants contributed to this cluster.

#### Category 2.1: Feeling prioritized in a non‐abusive therapeutic alliance was experienced as helpful, whereas a reserved therapeutic stance led to confusion and disappointment

A secure therapeutic relationship attuned to their needs was crucial for the interviewees to engage in therapy. Some felt ‘held’ (P4) or ‘caught’ (P8) by the therapist and valued being recognized for their accomplishments, despite traumatic histories. Many valued being heard without blame, especially in contrast to being blamed for their traumatic experiences in their private lives: ‘A long‐cherished dream has really come true for me! … That I have someone who listens to me … and who doesn't blame me for it’ (P6). Some participants found safety in the therapist's ability to tolerate distressing content, pointing out the fine line between empathy and overinvolvement: ‘I think I saw him swallow hard once or twice. And … that was okay. But it shouldn't have been any more than that, because then I wouldn't have told him anything more’ (P7).

Although some patients initially preferred a female therapist due to past abuse by men, two who were assigned male therapists reported unexpectedly positive encounters: ‘It was good for me to see…a young man…is very trustworthy and can really help me. … [that] there are many men who are different from the men I've experienced’ (P4). Experiences contrasting with previous relationship patterns also emerged when patients were given control over the pace of therapy, deciding what to discuss and having the option to pause when needed:

Well, he [the therapist] let me decide for myself. … I think, in that moment it was very, very important for me. … although it was hard for me … Because, I was just always used to being told what to do. (P6)

At the same time, patients emphasized that giving control should not lead to therapist passivity. Attuned therapists who monitored patients' emotional states and intervened when needed were experienced as supportive: ‘I go on and on… and [it was important] that she stopped me, because otherwise I decompensate’ (P2).

However, when therapists were perceived as too passive and emotionally unavailable, patients felt frustration and disappointment:

At some point … I said to him: ‘It would be nice if you cleared your throat every now and then, then I know that you are still here.’ And then he said: ‘Was that all too little for you?’ And then I said: ‘Yes, so when there is no feedback at all, then I don't know. … I need to know that you are available’. (P7)

Several patients expressed the wish for more active encouragement when disclosing their traumatic experiences. One participant hoped to speak about her trauma earlier in therapy and for the first time, and felt that the therapist did not encourage her enough to do so:

That she [the therapist] puts a bit more pressure and somehow pushes you, like in that direction, so that you vocalize it. I would have wished for more of that. Because … I … maybe could have done it a bit sooner with her. (P1)

Another participant found it difficult to talk about herself in therapy. At times, however, she had the impulse to speak explicitly about her traumatic experiences and felt confused and disappointed when her therapist held her back: ‘I was surprised that often, when I somehow felt I had to talk about certain situations, I was told that we didn't talk specifically about certain traumatic experiences’ (P3). She further noted that the therapy felt superficial because it focused predominantly on the current impact of trauma rather than on the traumatic experiences themselves.

#### Category 2.2: In light of the patients' complex trauma histories, therapy was considered too short

Most interviewees felt that 24 sessions were not enough, and termination was experienced as abrupt and painful. For many, the limited timeframe collided with their gradual development of trust. Several participants highlighted needing considerable time to open up, shaped by past trauma and relational difficulties, negative therapy experiences or no experience with therapy at all. One participant was initially ‘beating around the bush’ and concluded: ‘Opening up takes time. We probably could have achieved more if I hadn't been so scared’ (P7). Half the participants reported that their difficulties expressing themselves interfered with the therapy process. Some felt intimidated and were afraid of being judged: ‘So for me, it's always in my head, what does the person think now? Are they going to think badly of me now … and that has hindered me so much in therapy’ (P1).

Although most patients developed trust and deeper engagement over time, for half of them therapy ended mid‐process, reactivating the feeling of being abandoned. One patient noted:

For me, it was almost like a shock at the end when I realized, oh my God, … I felt like I just found a right starting point and then it ended, or the end was near. That really hit me. … I thought great, now you are just being left alone. (P8)

Another felt ‘like a dog being thrown out of a moving train’ (P7). Some participants found it helpful when the last sessions focused on reviewing achievements or central issues. Others experienced the option of follow‐up sessions or further treatment as helpful to cope with the termination. Overall, most patients felt that 24 sessions were not enough to address the extent of their traumatic history, expressed a desire to continue therapy or acknowledged the need for further treatment:

The time is simply too short. … but … I don't even blame you. There's just way too much that had happened in my life. And I just can't … work through it in such a short period of time. (P6)

#### Category 2.3: External life stressors could not be integrated into trauma work, thereby hindering progress

Most participants reported external stressors that worsened their well‐being, such as interpersonal conflicts, a friend's cancer diagnosis, or triggering environments. These made it difficult to maintain focus in therapy. Furthermore, these external life stressors could not be adequately connected to the participants' trauma‐related difficulties or approached within the overarching therapeutic goals, leaving participants feeling unable to give sufficient attention to the traumatic material. For example, one participant described how managing stressful parenting situations left little room for the intended therapy content: ‘The current [topics] often suppressed what we actually wanted to continue talking about from the previous week … and we couldn't really do that, because there were such extreme current problems which we somehow had to deal with’ (P4).

#### **CORE CATEGORY** **:** **Unfinished Business—The Challenge of Navigating** **Relational Trauma within a Limited Timeframe**

**Within a secure therapeutic relationship, patients took a risk and turned towards their trauma which brought relief and fostered a sense of hope that progress was possible. However, this very engagement with the traumatic past also led to new emotional challenges. Long‐standing issues, a reserved therapeutic stance, and the limited timeframe hindered further progress. Ultimately, patients could only begin to lift the burden of their relational trauma.**

A sense of relief emerged as patients developed greater capacity to confront and cope with their traumatic experiences, with a safe and needs‐oriented therapeutic relationship as a key factor. At the same time, the weight of newly accessed traumatic memories introduced emotional challenges which could not be worked through due to time limits. This ambivalent trajectory was reported by all patients in one way or another and illustrates the dynamic process of relief and distress through emotional depth when engaging with trauma. Patients learned to observe and regulate their emotions, which led to greater control and greater life satisfaction (Cluster 1: Categories 1.1 and 1.2). This progress was facilitated by their courage to engage more deeply with their trauma and by feeling adequately supported within the therapeutic relationship (Cluster 2: Category 2.1). At the same time, they also came into contact with trauma‐related memories and feelings, which led to a worsening of symptoms or the emergence of other distressing reactions (Cluster 1: Category 1.3). To consolidate their progress and integrate the traumatic experiences, including the re‐emergence of painful memories and emotions, patients would have needed more active and especially longer‐term therapeutic support (Cluster 2: Categories 2.1, 2.2 and 2.3). We chose the term ‘Unfinished Business’ to describe this dialectical dynamic in which therapeutic progress coexists with heightened vulnerability, symptom intensification or emotional destabilization. Rather than suggesting linear resolution, this dialectic reflects the paradox that opening up can both heal and unsettle, particularly in individuals with relational trauma. In this sense, the therapeutic process remains meaningful, yet incomplete.

## DISCUSSION

This study qualitatively explored how individuals with PTSD‐CM who showed no or only little improvement in their PTSD symptoms according to CAPS‐5 experienced change after TF‐PDT and the factors that facilitated or hindered it. This exploration provides a more nuanced understanding of these experiences. While the quantitative data identified participants who did not show clinically significant symptom reduction according to CAPS‐5, a central finding from qualitative data revealed that these participants nevertheless reported meaningful therapeutic insights and gains, such as improved emotional regulation and relational functioning. However, these subjective gains often co‐occurred with distress due to reactivated traumatic memories and feelings. While a secure therapeutic relationship attuned to the participants’ needs was perceived as essential for engagement, relational and temporal limitations as well as external life stressors hindered the integration of these changes, potentially explaining the absence of measurable improvement.

Our findings align with Carrington et al. (2024), who contextualize non‐response as ‘at the center of a continuum that ranges from improvement to worsening’ (p. 8). Extending this perspective, we observed a dialectical pattern in which moments of progress (Categories 1.1 and 1.2) often *co‐occurred* with symptom intensification (Category 1.3). This dynamic might be a typical characteristic in trauma‐related disorders: Although TF‐PDT generally limits regression and only one participant reported explicit trauma confrontation, associative reactivation (Wöller, 2016) may still evoke trauma‐related memories and feelings, leading to temporary destabilization. This might reflect a normal and temporary treatment effect rather than failure (Lindegaard et al., 2024; Linden & Schermuly‐Haupt, 2014). In fact, such destabilization can prompt development by disrupting rigid internal structures (Saketopoulou, 2019, 2022) as reflected in our participants’ accounts of gaining new emotional access and beginning to transform their traumatic narratives. While prior studies on numerical non‐response in depressed patients identified core categories centering on stagnation (De Smet et al., 2019; Werbart et al., 2015), our participants’ narratives pointed to an unfolding process of transformation. This indicates that individuals defined as ‘non‐responders’ may experience different processes depending on their primary clinical presentation.

Participants in our study reported improved self‐awareness, relational functioning and symptom management. In the context of trauma, participants reported developing a more nuanced understanding of themselves and their traumatic experiences, as well as a sense of empowerment through increased self‐awareness and clearer personal boundaries, echoing findings by Lepistö et al. (2025). Central to these gains was the therapeutic relationship. Participants in our study emphasized the importance of a safe, trusting bond which contrasted with prior abusive and neglectful relationships. The ability to disclose trauma without burdening the therapist was described as curative, and responsiveness to individual needs, an important component of trauma‐related therapies (Norcross & Wampold, 2019), was experienced as essential. These results are consistent with prior research on the helpful therapeutic alliance (Bordin, 1979), which was associated with improvement (Ellis et al., 2018; Howard et al., 2022), less dropout (Sijercic et al., 2021) and meaningful engagement (Dawood et al., 2025; Boterhoven de Haan et al., 2021; Parry & Simpson, 2016) in trauma‐related therapies. Conversely, when therapists were perceived as distant or unresponsive, participants were disappointed and disengaged. In Lepistö et al. (2025), participants reported losing motivation to meaningfully engage in treatment because of increased posttraumatic symptoms. In our study, symptom stagnation may have both stemmed from and contributed to a weakened alliance, creating a vicious cycle that hindered progress.

Participants in our study experienced initial mistrust and found it difficult to open up, which likely reflected past betrayals and assaults in close relationships (Dalenberg, 2004). Such patterns may manifest as reenactments of prior relational trauma within the therapeutic setting (Parry & Simpson, 2016). Therapists' reserved stances and limited encouragement of trauma disclosure may have reflected countertransference reactions to trauma content. In the context of PTSD‐CM, such reactions can range from emotional withdrawal and avoidance to overprotection (Cavanagh et al., 2015; Boterhoven de Haan et al., 2021; Wöller et al., 2020). If not managed or unnoticed, these reactions can lead to empathic strain (Wilson & Lindy, 1994) and have been associated with poor outcomes (Abargil & Tishby, 2022; Hayes et al., 2018). In some cases, therapists' emotional withdrawal may have served as a defense against perceived treatment failure (Carrington et al., 2024), potentially intensified by trial constraints and performance pressure (Marin et al., 2025).

Consistent with other studies (e.g., Carrington et al., 2024; Dawood et al., 2025; Boterhoven de Haan et al., 2021), participants in our study consistently criticized limited therapy duration or the perceived premature ending, which reactivated feelings of abandonment—a common experience in relational trauma (Pearlman & Courtois, 2005). Our sample included older individuals with up to five comorbidities, factors associated with non‐response in PTSD in previous research (Semmlinger et al., 2024). Although we did not investigate whether these characteristics were unique to individuals in our study, our qualitative analysis indicated that they required time to build trust, while facing both internal and external challenges throughout treatment. Such cases may require longer‐term treatment, as proposed by Wöller et al. (2020).

Ultimately, participants described meaningful changes that were not reflected in the external label of ‘non‐responder’. Instead, our study suggests that an important therapeutic process had begun, characterized by a dynamic interplay of emerging improvement and emotional distress that may be typical of trauma‐focused therapies. Such developments remain invisible when change is conceptualized solely as a shift in pre–post‐symptom scores, reducing therapeutic success to numerical differences within a limited timeframe and overlooking ongoing, subjectively meaningful processes. Quantitative ratings of change can further obscure the broad spectrum of individual trajectories that may themselves be ambiguous, contradictory, or opaque (Steinert, 2025). Our findings may also call into question what lies behind the high non‐response rates in individuals with PTSD: namely, a multifaceted process of simultaneous improvement and deterioration, one that has begun but may require more time.

### Strengths and limitations

Based on one of the largest RCTs in this field, this study offers novel insights into how particularly vulnerable individuals, individuals with PTSD‐CM, who— according to external quantitative measures did not respond to therapy— experienced therapeutic change. Several limitations must be acknowledged. Because the interviews had already been conducted in the context of a larger study, we could not adapt the interview questions for the purposes of our study or employ theoretical sampling (Levitt, 2021). Consequently, as our study deviated in one decisive aspect from a full GT analysis, the results may not capture the full theoretical framework typically sought in GT. Although we did not include all TF‐PDT ‘non‐responders’ interviewed in PP in our analysis, the cross‐check did not provide new information, suggesting that saturation was likely reached. Also, we could not contact participants for feedback on our results since this was not covered by our initial ethics approval. Dropouts who may hold critical perspectives on non‐response (Leichsenring et al., 2019; Lindegaard et al., 2024) were not interviewed. Since only a few negative experiences were reported, this may reflect participants’ discomfort with criticizing therapy, a dynamic identified among ‘non‐responders’ (Carrington et al., 2024). It is possible that participants felt safer criticizing the study context than the therapy itself (Fechau et al., 2024), potentially shaped by relational dynamics common in complex trauma. Also, the all‐female sample restricts generalizability to male individuals, a group overrepresented in PTSD non‐response (Semmlinger et al., 2024), thus limiting our ability to explore gender‐specific processes. Finally, as we defined non‐response solely by CAPS‐5 symptom reduction right after treatment, we cannot account for experiences of ENHANCE‐participants who numerically did not improve at follow‐up assessment or according to secondary outcomes such as emotion regulation, mentalization, or attachment (Leichsenring et al., 2020).

### Implications for future research and clinical practice

To capture a broader therapeutic process, future studies on PTSD‐CM should incorporate broader outcomes next to symptom reduction, including well‐being and life satisfaction (Schnurr et al., 2006). Also, further research should consider a broader definition of non‐response, including secondary outcomes such as emotion regulation. Mixed‐method case studies comparing those who benefited from therapy and those who did not, alongside therapist perspectives (Werbart et al., 2019) and different therapeutic approaches (Aardal et al., 2025) for PTSD‐CM, could illuminate divergent processes and distinct pathways to non‐response, respectively. In health care systems like Germany's, 24 sessions are classified as short‐term, while those exceeding 24 sessions are considered long‐term. Given the chronicity and complexity of PTSD‐CM, along with the time needed to build trust and address entrenched relational patterns, there is an urgent need for funding of trials which evaluate long‐term treatments or therapy durations tailored to individual needs.

Clinically, alliance‐building and opportunities for patients' feedback should remain central throughout therapy (Wöller et al., 2020). Given the relational nature of trauma, the therapeutic relationship should be seen as a core component of cure (Norcross & Wampold, 2019). While granting autonomy and control is essential (Dawood et al., 2025; Parry & Simpson, 2016; Wöller et al., 2020), therapists are encouraged to provide structure and responsive guidance. Participants in our study and in other studies (e.g., by Li et al., 2024) emphasized that treatment should feel responsive, not rigid. As therapist flexibility in applying interventions was associated with better outcomes (Owen & Hilsenroth, 2014), therapists should adapt treatment and interventions tailored to patients' needs. Trauma confrontation and trauma disclosure should never be forced, but neither should they be avoided; therapists should actively support their timing and application. Also, even without explicit trauma exposure, memories and feelings may resurface and require containment. Therapists should be aware of possible emotional responses and address them in supervision. Finally, therapists should also attend to external stressors and triggering environments, which are common among survivors of CM and may hinder therapeutic progress (Onsjö et al., 2025). Social support is a key protective factor in chronic PTSD (Steinert et al., 2015) and should be actively addressed in treatment. In our study, some of the participants' progress was clearly constrained by external stressors, highlighting the importance of integrating external realities into manualized treatment planning.

## CONCLUSION

This is the first qualitative study to examine how individuals with PTSD‐CM who did not show significant improvement in terms of symptom severity (CAPS‐5) experienced TF‐PDT. Our findings highlight the value of employing qualitative approaches from the participants’ perspective for capturing the complexity of therapeutic change and caution against equating ‘numerical non‐response’ (i.e., no symptom improvement) with therapeutic failure. Participants reported meaningful relational and emotional gains, even alongside symptom intensification, and these seemingly paradoxical developments became visible only through a qualitative design which provided the depth needed for these layers to emerge. These results underscore the importance of integrating patient perspectives and therapeutic processes into how we define and evaluate treatment success in complex trauma.

## AUTHOR CONTRIBUTIONS

**Fatima Nöske:** Conceptualization; writing – original draft; data curation; formal analysis; methodology; visualization; project administration; validation. **Frederike Döring:** Conceptualization; formal analysis; methodology; project administration; writing – review and editing. **Manfred E. Beutel:** Writing – review and editing; resources; project administration. **Melissa Hitzler:** Writing – review and editing; project administration. **Jürgen Hoyer:** Writing – review and editing; resources; project administration. **Elena Kabbathas:** Conceptualization; writing – review and editing; data curation. **Christine Knaevelsrud:** Writing – review and editing; resources; project administration. **Iris‐T. Kolassa:** Writing – review and editing; resources; project administration. **Johannes Kruse:** Writing – review and editing; resources; project administration. **Falk Leichsenring:** Conceptualization; writing – review and editing; resources; supervision; funding acquisition; validation; project administration. **Nina Marin:** Conceptualization; writing – review and editing; methodology; formal analysis; project administration. **Helen Niemeyer:** Writing – review and editing; project administration. **Simone Salzer:** Writing – review and editing; resources; project administration. **Karoline Sauer:** Writing – review and editing; project administration. **Marie Siebert:** Methodology; conceptualization; investigation; data curation; writing – review and editing; formal analysis; project administration. **Rudolf Stark:** Writing – review and editing; resources; project administration. **Visal Tumani:** Writing – review and editing; supervision. **Kerstin Weidner:** Writing – review and editing; resources; project administration. **Jörn von Wietersheim:** Writing – review and editing; resources; project administration. **Christiane Steinert:** Conceptualization; methodology; validation; resources; writing – review and editing; supervision; project administration; funding acquisition; formal analysis. **Wolfgang Wöller:** Writing – review and editing; conceptualization; supervision.

## FUNDING INFORMATION

The study Patient Participation (funding code: 1KR1801D) is embedded in the ENHANCE trial (funding code: 01KR1801A), both funded by the German Federal Ministry of Education and Research. The funder was not involved in the conceptualization of the study, data collection, data analysis and interpretation of results.

## CONFLICT OF INTEREST STATEMENT

The authors whose names are listed immediately below certify that they have no affiliations with or involvement in any organization or entity with any financial interest (such as honoraria; educational grants; participation in speakers' bureaus; membership, employment, consultancies, stock ownership, or other equity interest; and expert testimony or patent‐licensing arrangements), or non‐financial interest (such as personal or professional relationships, affiliations, knowledge or beliefs) in the subject matter or materials discussed in this manuscript.

## Supporting information


Data S1.

## Data Availability

The interview material (audios and transcripts) cannot be shared due to data protection reasons.

## Footnotes

^1^
In quantitative outcome studies on PTSD, non‐response is usually defined as failure of meeting the predefined response criteria, for example, pre–post change less than 30%–50% on the Clinician‐Administered PTSD Scale (CAPS, Varker et al., 2020). Broader definitions include lack of reliable or clinically significant symptom reduction despite treatment (Carrington et al., 2024).
