# Supplementary material for: Unfinished business: A grounded theory analysis of change among individuals classified as numerical non‐responders to psychodynamic psychotherapy for post‐traumatic stress disorder related to childhood maltreatment
Source: Psychol Psychother. 2026 Feb 18;99(2):621–44. doi: 10.1111/papt.70040 (PMC13162179; doi:10.1111/papt.70040)
Supplement: Supplementary file 1 — Data S1. [file PAPT-99-621-s001.docx]

**Client Change Interview (CCI) Protocol***
Adapted version based on Elliott (1999) and Elliott & Rodgers (2008)

Pat.-ID ___________________

Date ___________________

Interviewer ___________________

Project/Study ___________________

**General Information for Interviewers**

**Background**
Upon completion of therapy, patients are invited to participate in a semi-structured interview of approximately one to one and a half hours in duration. The main topics of this conversation are the changes patients have noticed since the start of therapy, the reasons they believe led to these changes, and helpful or unhelpful aspects of the therapy. Another topic concerns their experiences related to participating in the study.

**Optional (for part of the sample)**
One week before the interview, patients may receive a copy of the interview guide so that they can reflect on it in advance.

**Interview Strategy**

This interview works best as a loosely structured, empathic exploration of patients’ experiences with their therapy and study participation. As the interviewer, your role is to help patients tell the story of their therapy and study experience. This is best achieved through a curious, engaged attitude, using open-ended questions and empathic responses to encourage patients to share their experiences.
Given this, each question should first be asked in an open and minimally structured way. Only add structure where necessary. For some questions, alternative phrasings are suggested, which you may use but are not required to.
Instruct patients to describe their experiences as detailed as possible. Use the “what else?” prompt (e.g., *What else comes to mind? How else would you describe it?*)—without being too demanding and only until the patient has nothing further to add.

**Begin the Interview with an Introduction for Patients**

The aim of this conversation is for you to tell us—in your own words—about your therapy and your participation in the study. This information will help us better understand the mechanisms of therapy (or: better understand how the therapy works) and will also help us improve the therapy. As stated in the consent form, this conversation will be audio recorded for later anonymized transcription. Your therapist will not have access to this conversation. The transcripts will be analyzed anonymously.

Please provide as many details as possible for each aspect I ask about.

Do you have any questions before we begin? (Ensure that the person is in a safe and calm state before starting.)

**1. General Questions:** [approx. 5 min]
**1a. How are you doing now in general?**
**1b. How was the therapy for you? / How did it feel to be in treatment?**

**2. Changes:** [approx. 10 min]
**2a. Since the start of therapy, have you noticed any changes in yourself? If so, what changes are these?**

Interviewer**:** Discuss the changes with the patient and note them for later. If helpful, you may use some of these follow-up questions:

- Do you behave, think, or feel differently now than before therapy?
- What specific ideas have you taken away from therapy, including about yourself or other people?
- Have others noticed or commented on any changes?

**2b. Since the start of therapy, has anything changed for the worse?** (Has anything else changed for the worse?)

**2c. Is there anything you wanted to change that hasn’t since the start of therapy?** (Can you think of anything else you would have liked to change?)

**3. Evaluating the Changes** [approx. 10 min]
*(Go through each previously noted change)*
**3a.** **You’ve told me about several changes** (repeat changes briefly). **Were these changes surprising to you, or had you expected them in this or a similar form?**
3b. **How likely do you think it is that these changes would have occurred without the therapy?
3c. How important or significant is this change to you personally?**

**4. Attributions** [approx. 5 min]
**What do you think caused the various changes you’ve described? In other words: What do you think may have brought about these changes?** (This applies to factors outside as well as within the therapy.)

**5. Helpful Aspects** [approx. 10 min]
**All in all, what would you say was helpful to you in your therapy? Please give examples** (e.g., general aspects, specific events).

**6. Problematic Aspects** [approx. 5 min]
**6a. Which aspects of your therapy were more hindering, unhelpful, negative, or** **disappointing?** (e.g., general aspects, specific events)
**6b.** **Were there things in your therapy that were difficult or painful but still okay—or perhaps even helpful? Which ones?**
**6c. Was there anything missing in your therapy?** (What would have made your therapy more effective or helpful?)

**7. Significant Experiences** [approx. 5 min]
**Was there an experience that was particularly valuable or meaningful to you? If so, which one?**

**8. Resources** [approx. 5 min]
**8a. Which personal strengths do you think helped you make use of therapy to handle your problems?** (Skills, personal qualities)
**8b. Which aspects of your current life situation helped you make use of therapy to cope with your problems?** (Family, work, relationships, housing situation)

**9. Limitations / Difficulties** [approx. 5 min]
**9a. Which personal aspects do you think made it harder for you to handle your problems in therapy?** (Things about you as a person)
**9b. Which aspects of your current life situation made it harder for you to use therapy to cope with your problems?** (Family, work, relationships, living circumstances)

**10. The Study** [approx. 10 min]
**10a. What was it like for you to participate in this study?** (Initial screening, research interviews, filling out questionnaires, etc.)
**10b.** **Can you summarize what was helpful about participating in the study? Please give examples.**
**10c.** **Which aspects related to the study were hindering, unhelpful, negative, or interfered with therapy? Please give examples.**

**11. Suggestions** [approx. 5 min]
**Do you have any suggestions for us regarding the study or the therapy? Is there anything else you would like to share with me?**

**12. The CCI Conversation** [approx. 5 min]
**How was it for you to do this interview with me today?** (Interaction with the interviewer, style of conversation and questions, etc.)

**References**
Elliott, R. (1999) Client Change Interview Protocol. Available at: <http://www.drbrianrodgers.com/research/client-change-interview>. Accessed: 26.11.2021.
Elliott, R. & Rodgers, B. (2008). Client Change Interview Schedule (v5). Glasgow: University of Strathclyde.
